# Supplementary material for: Conserved subcortical processing in visuo-vestibular gaze control
Source: Nat Commun. 2022 Aug 10;13:4699. doi: 10.1038/s41467-022-32379-w (PMC9365791; doi:10.1038/s41467-022-32379-w)
Supplement: Supplementary file 1 — Supplementary Information [file 41467_2022_32379_MOESM1_ESM.pdf]

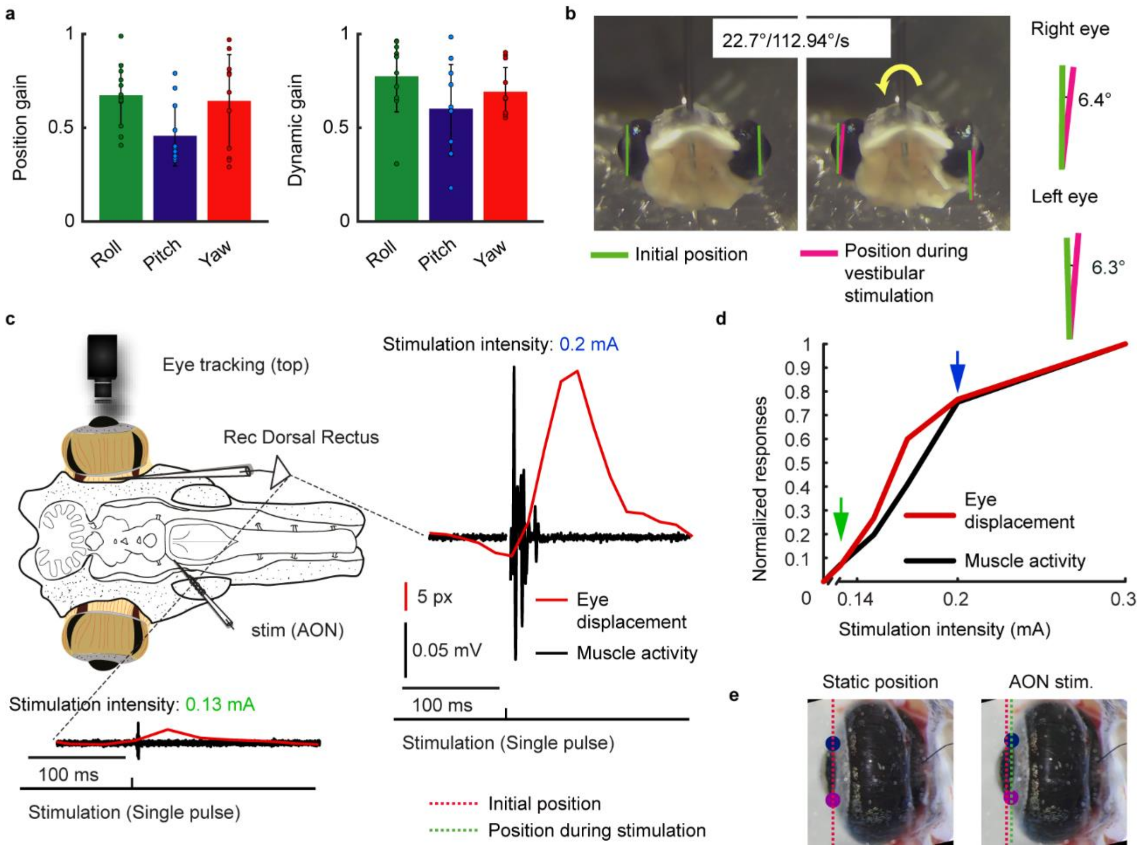

Supplementary Figure 1. VOR gain in lampreys, and EMG activity reflects eye movement activity. a, Plots representing the static (left) and dynamic (right) VOR gains after vestibular stimulation in the roll, pitch, and yaw planes (n = 12 slow phase events analyzed for each plane from three animals). Data are presented as mean values +/- SD. **b,** The *ex vivo* lamprey preparation, shown before and after a roll rotation to its right. Degrees between green and red lines signifies the eye movements response brought on by the vestibulo-ocular reflex. **c,** Representative traces showing EMG activity (black) superimposed with the displacement of the eye in the X axis (red), in response to electric stimulation of the anterior octavomotor nucleus (AON) at two different intensities (1.13mA, bottom; 0.2 mA, top-right). **d,** Graph showing EMG activity (black) and eye displacement (red) in response to increasing stimulation intensities of the AON. Both are normalized to the maximum value to allow comparison between them, showing that increasing levels of muscle activity correspond to larger eye movements. The arrows indicate the position in the graph for the shown traces, matching the

color of the stimulation intensity. **e,** Position of the eye before (left) and at the peak of electric stimulation of the AON at 0.2 mA (right). The red and green dotted lines mark the position of the same region in the eye before, and at the peak of the movement, respectively. The blue and purple dots indicate the position of the labels used to infer the trajectory of the eye. Source data are provided as a Source Data file.

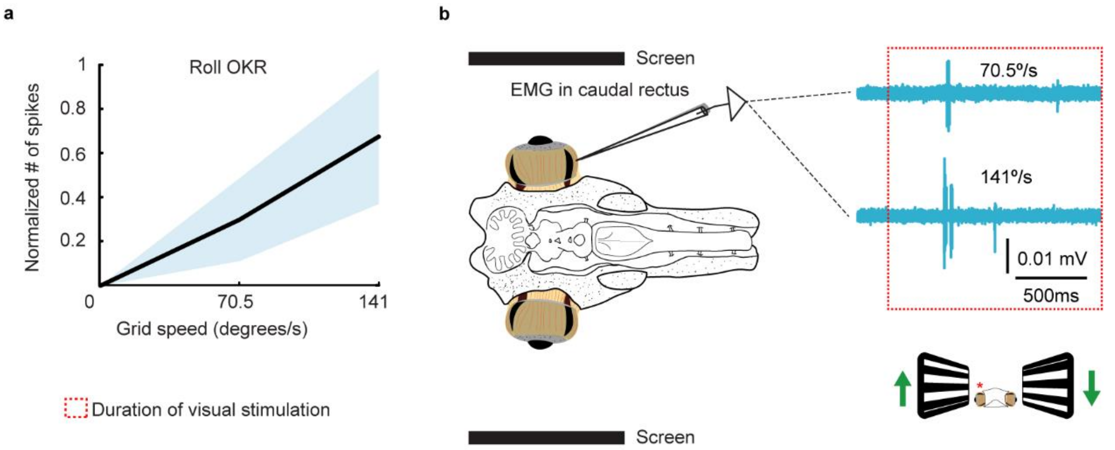

Supplementary Figure 2. Roll OKR is observed despite the lack of yaw OKR. a, Normalized number of spikes for the same animal showed in Fig. 2g-i (and which lacked OKR in the yaw plane) showing that reliable roll-OKR responses were recorded, as reflected in the increases number of spikes in parallel to stimulation speed. The shaded area denotes error bands. **b,** Raw traces from the dorsal rectus muscle of the same animal during optokinetic roll stimulations reflect increased OKR gain to increased velocities. The dotted rectangle indicates the duration of the visual stimulation. Source data are provided as a Source Data file.

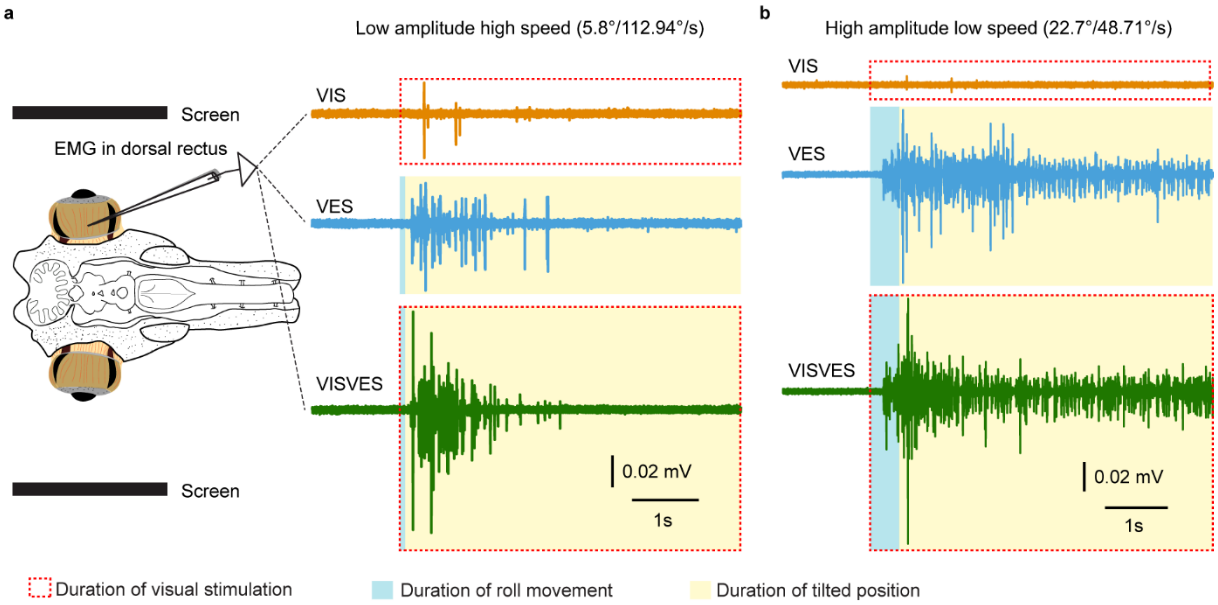

Supplementary Figure 3. Visuovestibular integration. a,b, Lamprey schematic outlining the preparation used during *ex-vivo* recordings (a, left). A recording electrode was placed in the right dorsal rectus, i.e. ipsilateral to rotation direction. To its right, representative traces for all three modalities, visual (VIS), vestibular (VES), and visuovestibular (VISVES) for the low amplitude-high speed roll stimulation (a) and high amplitude-low speed roll stimulation (b). The blue area indicates the duration of the roll movement, yellow the duration of static tilt, and the region delimited by the dotted red rectangle signifies ongoing optokinetic stimulation.

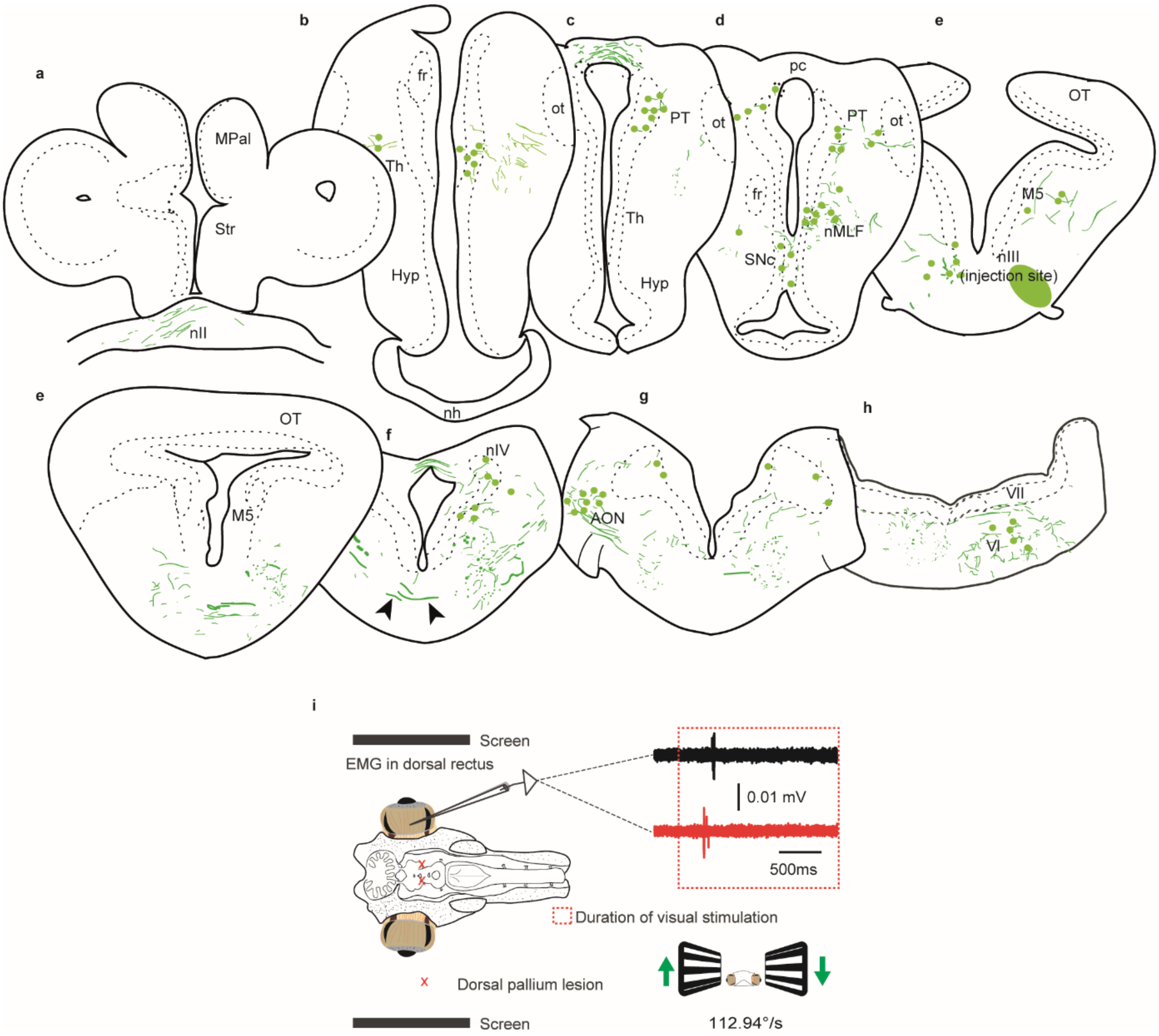

Supplementary Figure 4. Connectome of the oculomotor nucleus/Inactivation of the visual area in pallium does not abolish OKR. a-h, Connectivity of the oculomotor nucleus shown in representative schematic drawings of transversal sections from rostral to caudal. The injection site is shown in (e) indicated by a shadowed green area. Arrowheads show AON fibers crossing. **i,** The *ex vivo* preparation with flanking screens as seen from the top is presented to the left. In the top right are EMG responses in the dorsal rectus to a optokinetic stimulus in an intact brain (black trace) and after precise inactivation of the visual area in pallium through lesioning (red trace). Note that the visual response is maintained. The dotted rectangle indicates the duration of the visual stimulation. The illustration in the bottom right shows the preparation

from the front, with the green arrows indicating the direction of the bars in the optokinetic stimulation.

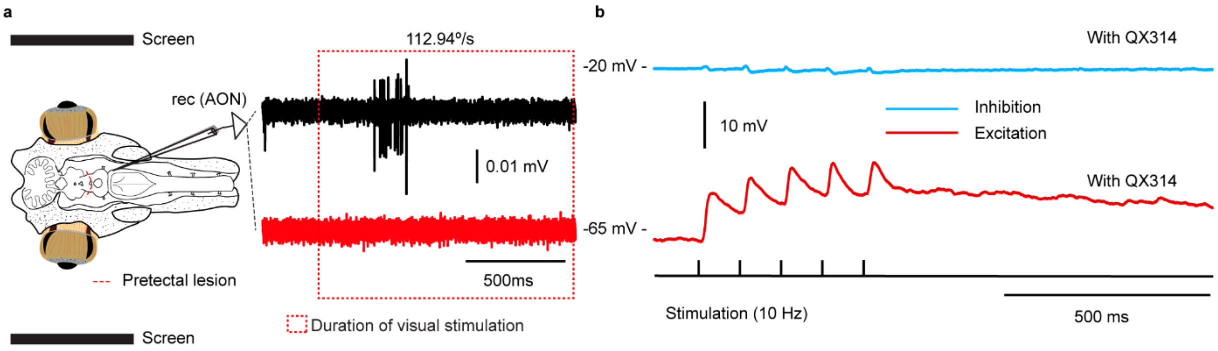

Supplementary Figure 5. Visual inputs to the vestibular area. a, Extracellular activity in the anterior octavolateral nucleus (AON) in response to an optokinetic stimulus in an intact brain (black trace), and after precise pharmacological inactivation of pretectum through mechanically lesioning this structure (red trace). Note that the visual responses to optokinetic stimulation are completely abolished. The location of the lesion and recording site is indicated in the schematic (left). The dotted rectangle indicates the duration of the optokinetic stimulation. **b,** Excitatory (bottom, red trace) but no inhibitory (top, blue trace) PSPs were evoked in prelabelled AON neurons projecting to the oculomotor nucleus in response to electric stimulation of the optic tract/pretectal region (10 Hz), with QX314 in the recording pipette to block spiking.

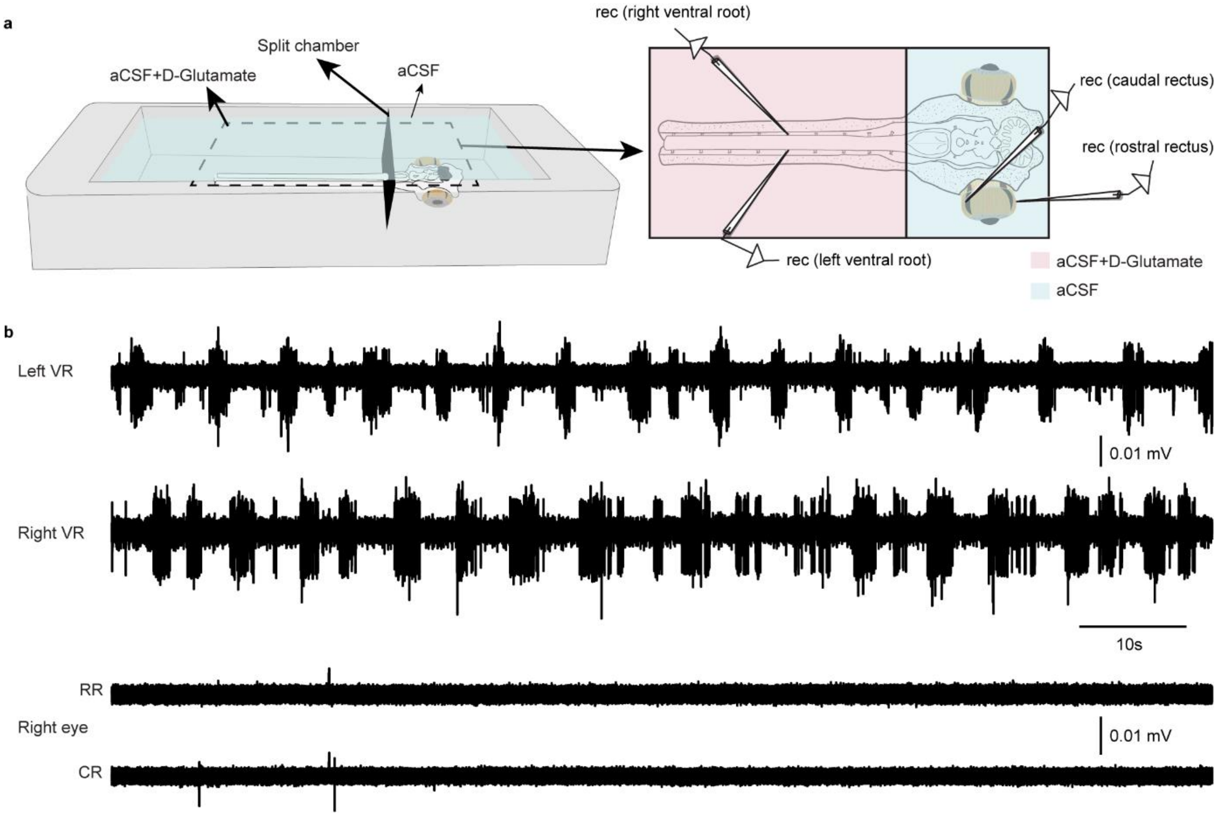

Supplementary Figure 6. Fictive locomotion does not result in coordinated extraocular muscle activity. a, A schematic to the left shows a split chamber allowing for separating the brain and the spinal cord in a lamprey *ex-vivo* preparation. This allowed the brain to be submerged in artificial cerebrospinal fluid (aCSF) while the spinal cord could be exposed to D-Glutamate mixed in the aCSF solution, initiating fictive locomotion. To the right is an illustration of the recording locations on the lamprey preparation while separated in the split chamber. Ventral root activity was recorded to monitor fictive locomotion, while both caudal and rostral rectus muscles were recorded to monitor possible movements in the yaw plane. **b,** Electrophysiological recordings from the electrodes in the ventral roots (VR) as well as in the rostral (RR) and caudal (CR) eye muscles. VR activity represents fictive locomotion as indicated by the alternating VR activity. No correlation to eye muscle activity was seen.
