# Supplementary material for: Conserved subcortical processing in visuo-vestibular gaze control
Source: Nat Commun. 2022 Aug 10;13:4699. doi: 10.1038/s41467-022-32379-w (PMC9365791; doi:10.1038/s41467-022-32379-w)
Supplement: Supplementary file 3 — Description of Additional Supplementary Files [file 41467_2022_32379_MOESM3_ESM.pdf]

Description of Additional Supplementary Files

File name: Supplementary Movie 1

Description: Representative eye movement in response to vestibular stimulation in the roll plane.

File name: Supplementary Movie 2

Description: Representative eye movement in response to vestibular stimulation in the pitch plane.

File name: Supplementary Movie 3

Description: Representative eye movement in response to vestibular stimulation in the yaw plane.

File name: Supplementary Movie 4

Description: Example of an experimental trial combining visuo-vestibular stimulation using our experimental platform.

File name: Supplementary Movie 5

Description: Representative trace (top) in response to combined visuo-vestibular stimulation (bottom).

File name: Supplementary Movie 6

Description: Eye movement in response to electric stimulation of the AON. The EMG recording of the dorsal rectus (black) is shown together with the trajectory of the eye (blue).

File name: Supplementary Movie 7

Description: Representative example of VOR slow and quick phases (right), in response to vestibular stimulation using a moving platform (left).

File name: Supplementary Movie 8

Description: Representative example of a lamprey swimming, where movements of the head can be perceived.

File name: Supplementary Movie 9

Description: Representative example showing eye movements coordinated with swimming. Top trace indicates the movement of one eye, while bottom trace indicates tail movements.
