# Supplementary material for: Conserved subcortical processing in visuo-vestibular gaze control
Source: Nat Commun. 2022 Aug 10;13:4699. doi: 10.1038/s41467-022-32379-w (PMC9365791; doi:10.1038/s41467-022-32379-w)
Supplement: Supplementary file 13 — Reporting Summary [file 41467_2022_32379_MOESM13_ESM.pdf]

nature portfolio

Corresponding author(s): Dr. Juan Pérez-Fernández

Last updated by author(s): Jun 25, 2022

Reporting Summary

Nature Portfolio wishes to improve the reproducibility of the work that we publish. This form provides structure for consistency and transparency in reporting. For further information on Nature Portfolio policies, see our Editorial Policies and the Editorial Policy Checklist.

Statistics

For all statistical analyses, confirm that the following items are present in the figure legend, table legend, main text, or Methods section.

n/a	Confirmed	
☐	☒	The exact sample size (*n*) for each experimental group/condition, given as a discrete number and unit of measurement
☐	☒	A statement on whether measurements were taken from distinct samples or whether the same sample was measured repeatedly
☐	☒	The statistical test(s) used AND whether they are one- or two-sided<br>*Only common tests should be described solely by name; describe more complex techniques in the Methods section.*
☐	☒	A description of all covariates tested
☐	☒	A description of any assumptions or corrections, such as tests of normality and adjustment for multiple comparisons
☐	☒	A full description of the statistical parameters including central tendency (e.g. means) or other basic estimates (e.g. regression coefficient) AND variation (e.g. standard deviation) or associated estimates of uncertainty (e.g. confidence intervals)
☐	☒	For null hypothesis testing, the test statistic (e.g. *F*, *t*, *r*) with confidence intervals, effect sizes, degrees of freedom and *P* value noted<br>*Give P values as exact values whenever suitable.*
☒	☐	For Bayesian analysis, information on the choice of priors and Markov chain Monte Carlo settings
☒	☐	For hierarchical and complex designs, identification of the appropriate level for tests and full reporting of outcomes
☐	☒	Estimates of effect sizes (e.g. Cohen's *d*, Pearson's *r*), indicating how they were calculated

Our web collection on statistics for biologists contains articles on many of the points above.

Software and code

Policy information about availability of computer code

Data collection	Matlab R2020b with the Psychtoolbox-3 extension was used to present visual stimuli, coordinated with Arduino 1.8 for controlling the platform servo motor. pClamp 10.2 was used to acquire the electrophyiological data.
Data analysis	SPSS 25, JASP 0.16, Matlab R2020b, DeepLabCut 2.2, Adobe Illustrator CC 2019, GIMP 2.1, Zeiss LSM software, ImageJ 1.7.

For manuscripts utilizing custom algorithms or software that are central to the research but not yet described in published literature, software must be made available to editors and reviewers. We strongly encourage code deposition in a community repository (e.g. GitHub). See the Nature Portfolio guidelines for submitting code & software for further information.

Data

Policy information about availability of data

All manuscripts must include a data availability statement. This statement should provide the following information, where applicable:

- Accession codes, unique identifiers, or web links for publicly available datasets
- A description of any restrictions on data availability
- For clinical datasets or third party data, please ensure that the statement adheres to our policy

Source data are provided with this paper and can be downloaded in the following link (https://doi.org/10.5281/zenodo.6628365), together with additional raw data. Further information and requests should be addressed to the corresponding author.

Human research participants

Policy information about studies involving human research participants and Sex and Gender in Research.

Reporting on sex and gender	*Use the terms sex (biological attribute) and gender (shaped by social and cultural circumstances) carefully in order to avoid confusing both terms. Indicate if findings apply to only one sex or gender; describe whether sex and gender were considered in study design whether sex and/or gender was determined based on self-reporting or assigned and methods used. Provide in the source data disaggregated sex and gender data where this information has been collected, and consent has been obtained for sharing of individual-level data; provide overall numbers in this Reporting Summary. Please state if this information has not been collected. Report sex- and gender-based analyses where performed, justify reasons for lack of sex- and gender-based analysis.*
Population characteristics	*Describe the covariate-relevant population characteristics of the human research participants (e.g. age, genotypic information, past and current diagnosis and treatment categories). If you filled out the behavioural & social sciences study design questions and have nothing to add here, write "See above."*
Recruitment	*Describe how participants were recruited. Outline any potential self-selection bias or other biases that may be present and how these are likely to impact results.*
Ethics oversight	*Identify the organization(s) that approved the study protocol.*

Note that full information on the approval of the study protocol must also be provided in the manuscript.

Field-specific reporting

Please select the one below that is the best fit for your research. If you are not sure, read the appropriate sections before making your selection.

☒ Life sciences ☐ Behavioural & social sciences ☐ Ecological, evolutionary & environmental sciences

For a reference copy of the document with all sections, see nature.com/documents/nr-reporting-summary-flat.pdf

Life sciences study design

All studies must disclose on these points even when the disclosure is negative.

Sample size	Sample sizes were chose based on previous protocols using the same animal model (Pérez-Fernández et al., 2017; Suzuki et al., 2019; Suryanarayana et al., 2020). For behavioral trials, three animals were used given the reliability of the obtained eye movements to minimize suffering. For experiments using the ex vivo preparation, the number of animals and repetitions for each trial within the same animal were chosen based on the significance of the statistical tests used through the study, and to ensure the viability of the preparation.
Data exclusions	In some cases, spontaneous eye movements were generated immediately before or after the stimulation, clearly affecting the responses analyzed. After visually inspecting putative outliers, a Grubb's test was used to identify significant outliers, and data points outside of the 95% confidence interval were removed prior to performing the statistical analysis. The total number of removed traces amounted to five for visuovestibular integration analysis, and two for time-to peak analysis after tectal lesioning.
Replication	While data was pooled for several recording per animal (usually three for each condition, to ensure the viability of the preparation), each variable was also tested for several preparations (whose number varied depending on the significance of statistical analysis). These are indicated as n for number of data points, and N for animal numbers. Responses were reliably reproduced between animals, with differences in signal strength being presented in the manuscript. For experiments in intact animals, we used three to six animals. For OKR and visuovestibular integration analysis, 5 to 13, depending on the significance of the obtained results. All lesion experiments were performed in at least three animals. Experiments using the semi-intact preparation were performed in 6 animals.
Randomization	The stimulation order was allocated by stratified randomization, i.e. each preparation was exposed to each stimulation type only once but in a random order. Samples were allocated randomly for all experiments.
Blinding	Blinding was not possible relevant for this study, as it required supervising the preparation at all times while controlling the independent variables. Additionally, we did not test any intervention effect, but rather compared physiological responses to pre-programmed stimulation that the tester had no control over.

Reporting for specific materials, systems and methods

We require information from authors about some types of materials, experimental systems and methods used in many studies. Here, indicate whether each material, system or method listed is relevant to your study. If you are not sure if a list item applies to your research, read the appropriate section before selecting a response.

Materials & experimental systems

n/a	Involved in the study
☒	☐ Antibodies
☒	☐ Eukaryotic cell lines
☒	☐ Palaeontology and archaeology
☐	☒ Animals and other organisms
☒	☐ Clinical data
☒	☐ Dual use research of concern

Methods

n/a	Involved in the study
☒	☐ ChIP-seq
☒	☐ Flow cytometry
☒	☐ MRI-based neuroimaging

Animals and other research organisms

Policy information about studies involving animals; ARRIVE guidelines recommended for reporting animal research, and Sex and Gender in Research

Laboratory animals	44 adult Lampetra fluviatilis and 4 young Petromyzon marinus acquired from authorized suppliers. Age is not provided by suppliers and therefore impossible to determine.
Wild animals	No wild animals were used in this study.
Reporting on sex	Both sexes were used throughout the study. Possible sex differences were not considered relevant given that the mechanisms analyzed (basic eye movements) should be the same for both sexes.
Field-collected samples	This study did not involve samples collected from the field.
Ethics oversight	The experimental procedures were approved by the local ethics committee (Stockholms Norra Djurförsöksetiska Nämnd) and the Xunta de Galicia under the supervision of the University of Vigo Committee for Animal use in Laboratory in accordance with the directive 2010/63/EU of the European Parliament and the RD 53/2013 Spanish regulation on the protection of animals use for scientific purposes. This information is included in the manuscript.

Note that full information on the approval of the study protocol must also be provided in the manuscript.
